# Supplementary figures and images for: Centrioles are amplified in cycling progenitors of olfactory sensory neurons
Source: PLoS Biol. 2020 Sep 15;18(9):e3000852. doi: 10.1371/journal.pbio.3000852 (PMC7518617; doi:10.1371/journal.pbio.3000852)

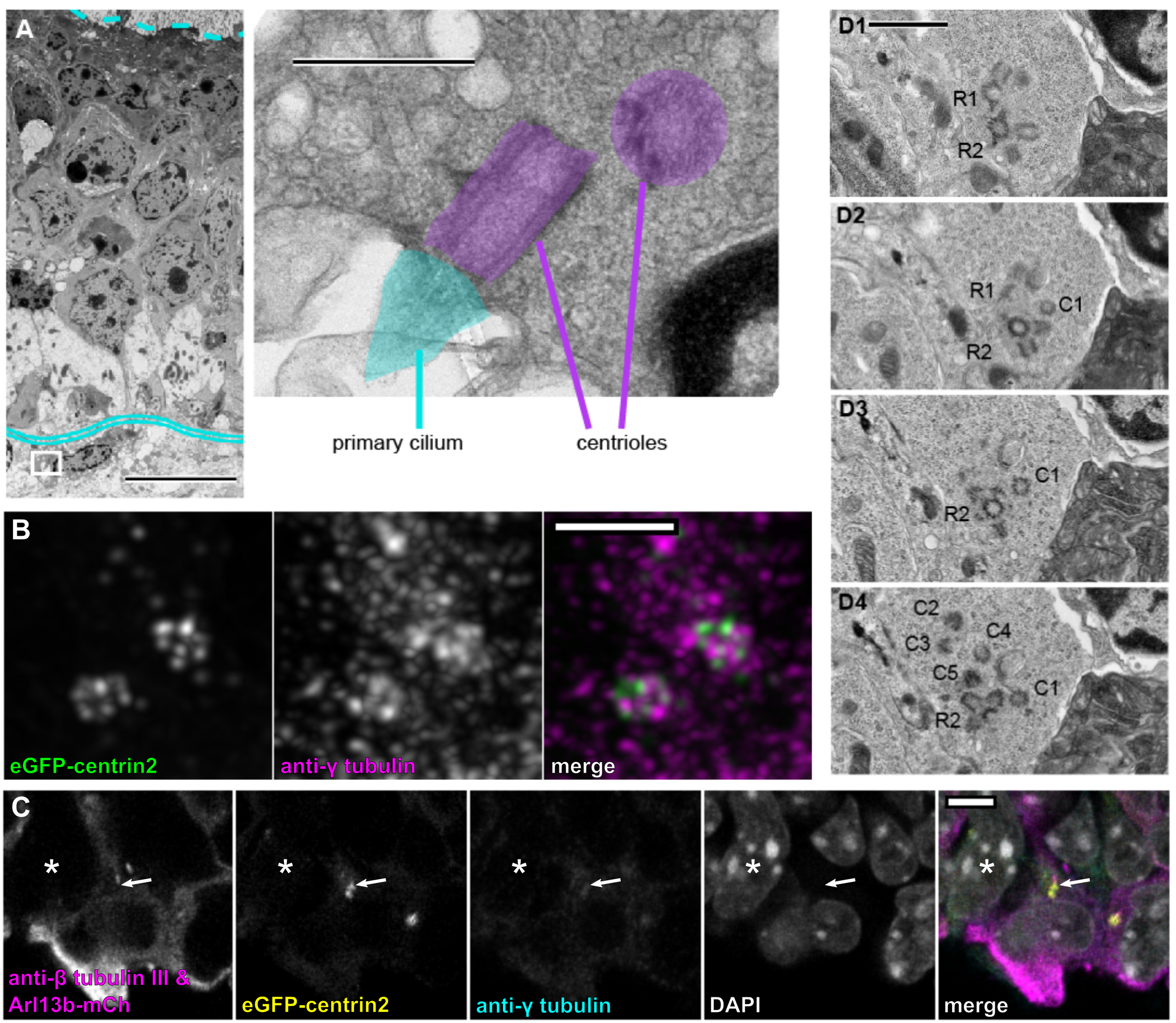

Supplement: S1 Fig — (A) TEM image of wild-type adult mouse olfactory epithelium. Dashed line marks the apical surface of the olfactory epithelium. Double solid line marks the basal lamina. Box marks the location and orientation of the inset shown in the panel to the right. Scale bar = 10 μm. The inset shows a centrosome and primary cilium pseudocolored purple and cyan, respectively. Inset scale bar = 0.5 μm. (B) Inset from a maximum projection fluorescence image of embryonic olfactory epithelium at E12.5 in mice expressing eGFP-centrin2 to mark centrioles, shown in Fig 1C. Deconvolved images show two rosette-like centriole clusters and separate puncta positive for centriole markers eGFP-centrin2 and γ tubulin. Scale bar = 2 μm. (C) Inset from a single optical section of embryonic olfactory epithelium at E12.5 in mice expressing eGFP-centrin2 to mark centrioles, shown in Fig 1C and S1B. Arrows mark the location of a rosette (shown in panel B) at the base of a primary cilium in a cell that is positive for β tubulin III. Asterisks mark a nearby cell that is negative for β tubulin III. Labels denote method of detection. Scale bar = 5 μm. (D1-D4) TEM images from serial sections of olfactory epithelium from a wild-type adult mouse. R1, R2 denote centriole rosettes, identified by morphology. C1-5 denote centrioles not associated with rosettes. Note that both mother centrioles in panel D1 have accessory structures, confirming that both rosettes form on centrioles that existed for at least one previous cell cycle. Scale bar = 1 μm. TEM, transmission electron microscopy. (TIF) [file pbio.3000852.s001.tif]

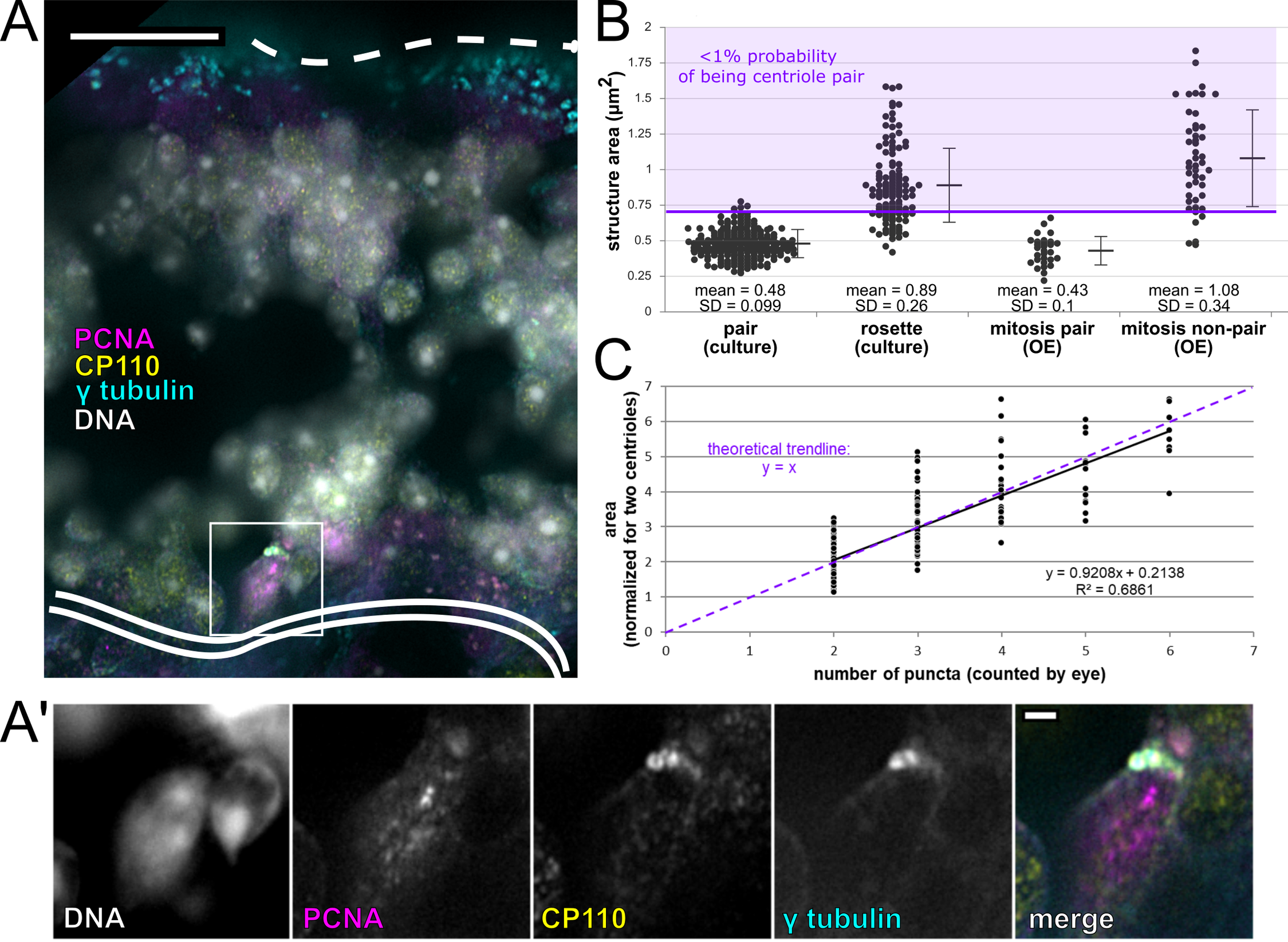

Supplement: S2 Fig — (A) Immunofluorescence in cryosections of olfactory epithelium from a wild-type adult mouse. Punctate nuclear PCNA marks a cell in S phase, whereas nearby nuclei are PCNA-negative. Dashed line marks the apical surface of the olfactory epithelium. Double solid line marks the basal lamina. Box marks the location of the inset. Scale bar = 20 μm. In the inset (A’), DAPI marks DNA of the S-phase cell, identified by punctate PCNA. CP110 marks the distal ends of centrioles and γ tubulin marks centrosomes. In this single optical section, daughter centrioles are visible as rings around γ tubulin foci, consistent with rosette formation. For clarity, the DNA panel is excluded from the merge. Inset scale bar = 2 μm. (B) Analysis of eGFP-centrin2 fluorescence area in mitotic cells in the olfactory epithelium. The pair (culture) column (N = 3, n = 208) shows measurements of centriole pairs in RPE-1 cells, which were used to set a threshold of 0.7085 μm2 (purple line), above which area measurements have <1% probability of belonging to the centriole pairs data set. The rosette (culture) column (N = 3, n = 115) shows measurements of centriole rosettes in cells overexpressing Plk4, 73.0% of which are above the threshold. The mitosis pair (OE) column (N = 5, n = 29) shows measurements of centriole pairs in adult olfactory epithelium, all of which fall below the threshold. The mitosis nonpair (OE) column (N = 5, n = 46) shows measurements of centriole structures which could not be definitively classified as pairs. A total of 87.2% are above the threshold. See S1 Data for measurement values. (C) Plot of anti-GFP fluorescence area against centriole number in cell culture. Immunofluorescence images were taken of hTert RPE-1 TetON-Plk4, eGFP-centrin2 cells with and without doxycycline induction. Anti-GFP fluorescence area of Sass6-positive structures was measured, and puncta were counted by eye. A line of best fit was generated in Microsoft Excel. The slope of the line is 0.9208, showing a [file pbio.3000852.s002.tif]

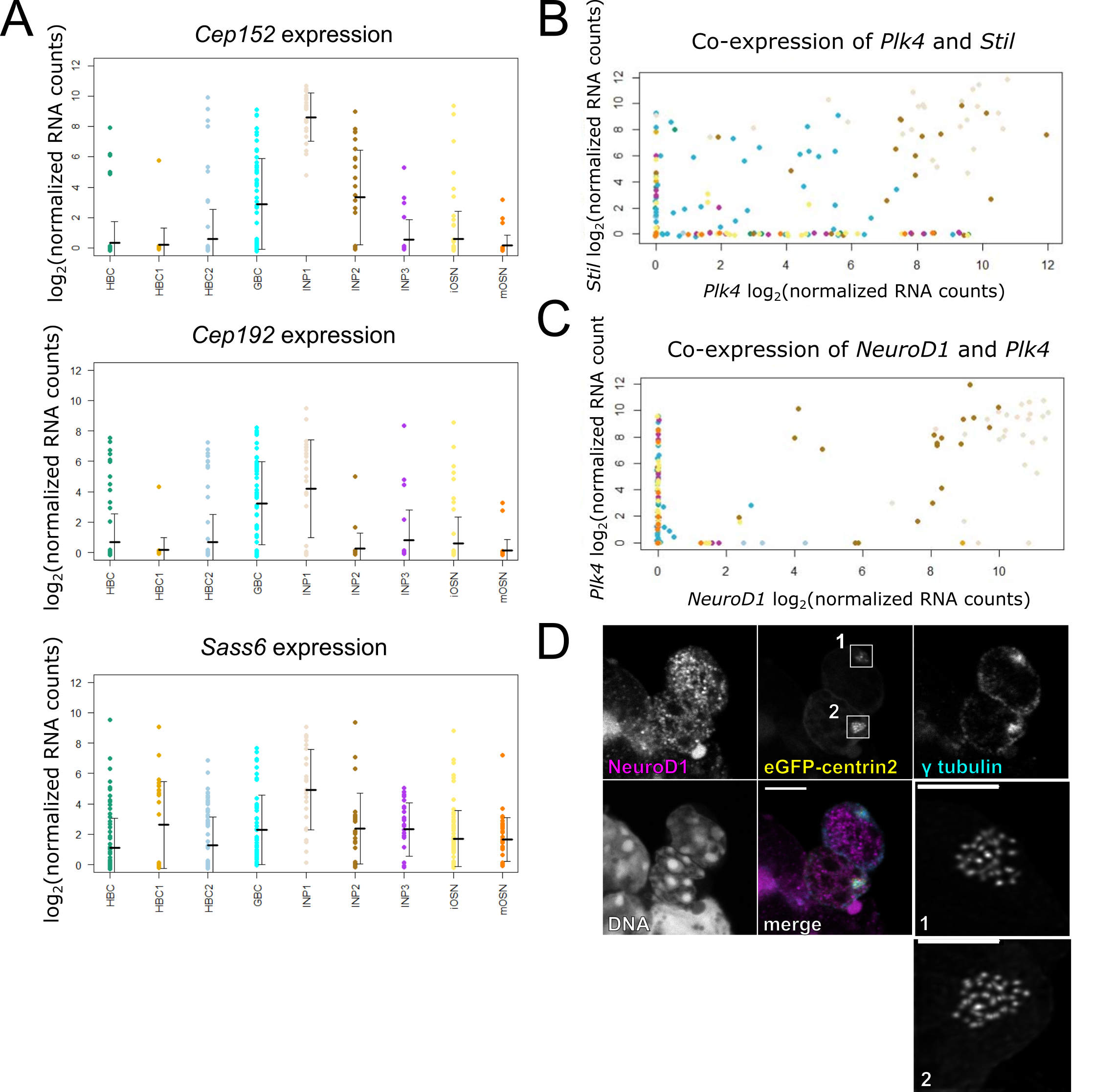

Supplement: S3 Fig — (A) Secondary analysis of an existing single-cell RNA sequencing data set from Fletcher and colleagues (2017) compares RNA levels for specific genes across cell types in the olfactory epithelium. The vertical axis shows log2(normalized RNA counts). Cell groups are ordered by pseudotime along the horizontal axis. Plots show RNA levels for Cep152, Cep192, and Sass6, genes required for centriole duplication (see Hatch and colleagues, 2010; Gomez-Ferreria and colleagues, 2007; Leidel and colleagues, 2005). Dots represent individual cells. Center lines = mean, and error bars = standard deviation. See S2 Data for values. (B-C) Secondary analysis of data from Fletcher and colleagues, 2017 shows coexpression of genes. Each dot represents a single cell. Both axes show log2(normalized RNA counts) (B) Plot shows RNA levels for Plk4 and Stil, centriole-associated genes known to drive rosette formation in cell culture. Points in the upper right corner are cells that express both genes at high levels. These are INP1 and INP2 cells (see A for color coding). (C) Plot shows RNA levels for NeuroD1, a transcription factor that marks INP1 and INP2 cells, and Plk4. Points in the upper right corner are cells that express both genes. These show that individual INP1/2 cells express high levels of Plk4. (D) A fluorescence image of NeuroD1-positive cells in dissociated olfactory epithelium. Note that this image includes other nuclei that are NeuroD1-negative. Boxes in the eGFP-centrin2 panel mark the locations of insets. DNA is excluded from the merge. Scale bar = 5 μm. The insets (1, 2) show deconvolved maximum projection images of centrioles, marked by eGFP-centrin2. Inset scale bars = 2 μm. Cep152, centrosomal protein 152; Cep192, centrosomal protein 192; eGFP, enhanced green fluorescent protein; INP, immediate neuronal precursor; NeuroD1, neuronal differentiation 1; Plk4, polo-like kinase 4; Sass6, spindle assembly abnormal protein 6; scRNAseq, single-cell RNA sequencing; Stil, SCL/Tal i [file pbio.3000852.s003.tif]
