# Supplementary material for: Centrioles are amplified in cycling progenitors of olfactory sensory neurons
Source: PLoS Biol. 2020 Sep 15;18(9):e3000852. doi: 10.1371/journal.pbio.3000852 (PMC7518617; doi:10.1371/journal.pbio.3000852)
Supplement: Attachment — Submitted filename: ChingandStearns_ResponseToReviewers.docx [file pbio.3000852.s007.docx]

**Response to Reviewers**

We thank the reviewers and editor for the positive and helpful comments on the manuscript. We have addressed the issues raised as follows:

Reviewer #1:

*I think it would be valuable to show some of the RNA seq data for the other key regulators of centriole biogenesis, such as SAS6 or CEP192. I understand these proteins do not change, but that might be good to show. It would also be interesting to know how many other genes show striking upregulation in these cell types*.

Thank you for the suggestion. We have now included analysis for Sass6 and Cep192 in figure S3A. We note that expression for both genes does show a modest increase in INP1 cells, relative to GBCs, however, this increased expression is less specific to these cell types, and the upregulation is of lower magnitude. This is part of the rationale for our choice to present Plk4 and Stil as a signature of centriole amplification timing, and thus is helpful to show.

*Is there any information on P53 levels/activity in the cells that have an amplified centriole content? Do the authors observe upregulation of P21 or other P53 target genes for example?*

This is an intriguing area to look in to, given the relationship between p53 status and altered centriole numbers that has been identified in previous work. We checked p53 and many of its known target genes; although some show differential expression in the dataset from Fletcher et al., 2017, no obvious patterns emerged. A more thorough investigation of the p53 pathway during and after centriole amplification in the olfactory epithelium would be an interesting follow-up to our work.

Reviewer #2:

*The paper explores an important topic and the findings could be interesting to the wide readership of PLOS biology. The drawback is that most imaging data is presented in a really poor quality. It is possible that the issue is only related to PDF presentation, but regardless of the reason, this issue impeded proper evaluation of the data.*

We thank the reviewer for this comment. We realized in preparation of the final figures for submission that those in the original manuscript were of lower resolution than they should have been. This has now been fixed. In addition, we do acknowledge that many images of the olfactory epithelium that we present here are less “clear” than typical images derived from cells grown on coverslips in culture (with which we have long experience). The reason for this is intrinsic to the nature of the experiments, which involve imaging cryosections of tissue from the mouse olfactory epithelium. In optimizing the imaging for these experiments, we found that a cryosection thickness of 14 μm best balances the need to capture most of the volume of a cell while still affording us the resolution to identify centriole number and configurations. A consequence of this constraint is that scattering of light through the tissue and relatively high background fluorescence reduce the image quality. The experiments referenced are also primarily ones in which we had to use wide-field microscopy to be able to scan for rare cells in the desired state. We are working on applying recent advances in light microscopy techniques, particularly expansion microscopy, to these samples but expect that optimizing these will take some time and are beyond the scope of the current work. Additional points regarding the images are addressed below.

*Point 1. Fig. 1C. beta tubulin III (a neuronal marker) and Arl13b (a ciliary marker) are both immunolabeled in the same color. Due to the poor image quality, it is difficult to understand which signal is which and why was this combination of markers used? Also, it is almost impossible to understand to which cell amplified centrioles belong to*.

We appreciate the complexity in this fluorescence image and thank the reviewer for pointing out this potential point of confusion. Arl13b is shown in the image because the mice express an Arl13b-mCherry construct in addition to the eGFP-centrin2 that was required for this imaging. We have found it useful to include both β tubulin III and Arl13b in our analysis to identify neuronally-fated cells and to provide additional evidence that a functional centrosome is present in the cell. These two markers were imaged in the same channel because Arl13b-mCherry is limited to the cilium, whereas β tubulin III stains the entire microtubule cytoskeleton. Furthermore, the presence of a single cilium in a rosette gives us greater confidence that centrioles from the apical surface weren’t sheared off and mis-localized during sample preparation (we have observed this in some cases), as OSN dendrites have many β-tubulin-III-positive cilia, in contrast to the single primary cilium here.

To clarify and aid readers in interpreting this image, we’ve added cropped and annotated panels from a single optical section to show that the rosette-bearing cell is positive for β-tubulin III, in contrast to nearby cells. This is now figure S1C, and references to this point have been adjusted accordingly in the text (line 94).

*Point 2. Fig. S2. Image quality is again a big issue. The content of the inserts in A' s is rotated with respect to the original low image panels in this figure (and throughout the manuscript on multiple places). This is not logical and could confuse readers. I would suggest that this is corrected*.

We have gone through the manuscript and oriented all low-magnfication views with the basal lamina down, and inset images so that they are in the same rotational position as the inset box drawn in the low-mag image. This is noted in the Methods section. Additionally, we’ve removed the DNA channel from the merge in figure S2 to make it easier to see the other markers, and we’ve increased the brightness of the DNA channel in the inset panel to make it easier to identify the position of the nucleus. We believe that, together, these changes will make it easier for readers to evaluate the data.

*Point 3. Figure 2 A: DNA condensation near the basal lamina is not obvious. Provide clearer images. Please keep the same orientation of centrioles in A and in A'*.

The deconvolved images for phospho-H3 and DNA are now included in figure 2A’, and the insets have been rotated to match the low-magnification image in figure 2A. The panels were also rearranged to accommodate this change.

*Point 4. Figure 2 B. It is unclear why was Poly E tubulin used. Or is it gamma tubulin as stated in the legend?*

This was an error in labeling, which we thank the reviewer for catching! The image actually shows staining for acetylated tubulin to show the mitotic spindle, and this has been corrected in the figure and its legend.

*Point 5. Figure 2 D. Based on a poor overlap between DNA and phosphor-H3 staining, it is not clear that the image represents anaphase. DNA is smeared all over the place.*

This point highlights the fact that our explanation of staging by phospho-H3 staining was insufficient. Phospho-H3 signal is expected to become dimmer and begin to relocalize away from chromatin during anaphase, as described in a review by Prigent and Demitrov (2003) and a study by Li et al. (2005). Unfortunately, anaphase chromosomes are difficult to clearly visualize in this tissue-imaging context, so we rely primarily on the phospho-H3 signal to interpret panel 2D. We have now included additional details and references in lines 132-135 and the figure 2 legend for how the stages of mitosis were identified, including that “Previous studies have shown that the signal for phosphorylation at serine 10 of histone H3 gradually increases to become brightest at metaphase, then decreases and relocalizes away from chromatin during anaphase [19,20].”

*The same comment can be addressed to Fig. 2 E. where the DNA pattern indicates that two nuclei are clearly out of mitosis and with decondensed DNA. Yet the cell boundaries drawn in white imply that these two nuclei are not yet separated (telophase?) and in the text (line 119 they are describes to be "later in division" (Fig. 2C, E-E').*

Again, our explanation was not thorough enough, and we thank the reviewer for highlighting this area for improvement. In panel 2E, the white cell boundaries are meant to show the formation of a cleavage furrow in either telophase or cytokinesis. At these stages, we expect to see DNA decondense, as the reviewer noted. Without additional markers, we can’t distinguish between telophase and cytokinesis, but we have clarified the legend of figure 2 to say, “DAPI shows DNA decondensing to form two daughter cell nuclei. White lines show cell boundaries, including a cleavage furrow, as approximated by cytoplasmic eGFP-centrin2 resulting from overexpression.” Lines 142-143 are similarly edited.

*Point 6. Abstract, Line 29. The authors suggest that the cells with amplified centrioles divide one or more times to become OSNs. But there is no evidence that these cells divide more than once. Would that imply that cells should have more than 2 mature centrioles? This should be experimentally tested if the authors think that it is a possibility*.

Our intent is to state that the cells can undergo a cell division without implying that the cells undergo exactly a single cell division, as we have no evidence against the occurrence of a subsequent cell division. This is particularly important in the olfactory epithelium, as the number of divisions might not be highly stereotyped in the pathway from basal progenitors to fully differentiated cell type (see lineage tracing in work from Fletcher et al. PMID: 28506465 and Gadye et al. PMID: 29174333). We have modified the sentence to say, “These cells then go on to divide at least once to become OSNs.”

*Point 7. The authors might want to revisit discussion. It feels disproportionally long and at times unnecessarily speculative. For instance:*

We agree that the Discussion section was relatively long, given the overall length of the manuscript. We’ve revised it to remove two of the paragraphs that included the most speculation and tangential information. Those that remain have been re-worded to address the points below.

*Line 176 - 198: The authors estimate that the number of centrioles formed per mother centriole is 8 and question how cells reach a greater and "desired" number of centrioles. I am not sure that I understand the argument about the "desired" number of centrioles in OSNs since mature OSNs harbor a wide range of centrioles in mature OSNs (6-37, line 75). So, the number of centrioles in mature OSNs seems random. It would also agree with the variable number of C1-GFP foci detected in various cells (Fig. 2A', E', E'', S3). In addition, it is evident that all Centrin-GFP signals are not in rosette-like configurations and adjacent to mother centrioles. This should not be surprising given that even in cycling cells after Plk4 overexpression the number of centrioles is random and their orientation with respect to mother centrioles is not always preserved. The authors also speculate that centrioles could undergo a second round of amplification, but this should be tested rather than speculated*.

We agree that the range of centriole number observed in OSNs is quite large, and this portion of the discussion is primarily concerned with the higher part of that range. A rosette with up to 37 centrioles was not observed. Hence, there is still a need to reconcile the OSN centriole numbers with the much smaller number of centrioles observed in rosettes, particularly by TEM. Although this expert reviewer may not be surprised to know that the amplification capacity of the rosette pathway could be higher than usually thought, this point is not widely known, and we feel that it is important to make clear to readers who might be less familiar with the field. Given the techniques used here, we could not exclude the possibility that centrioles are amplified further, after rosette formation and cell division. Live imaging, lineage-tracked centrioles, and pulse-chase are some possible techniques to experimentally test this possibility, but we feel that development and application of these tools would be beyond the scope of this work.

We have clarified the above points in what is now the second paragraph of the Discussion section.

*Line 200: The authors discuss possible detrimental consequences of centriole amplification on mitosis of progenitor cells. However, the presence of rosettes or de novo formed centrioles wont necessary lead to mitotic issues. Such issues usually occur if cells contain multiple mature centrioles (which are not detected in progenitor OSNs). The authors do not provide any data for or against possible mitotic issues in cells harboring amplified centrioles, yet, they discuss this is a great length and provide possible explanations why there might be no issues with mitosis*.

The participation of centrioles and centrosomes in the formation of mitotic spindle and their segregation on the spindle make this an important point of discussion. However, we agree that a lack of centriole-to-centrosome conversion is the likely explanation for how amplified centrioles will not interfere with spindle formation, with the caveat that this is only true if cells undergo no more than one division in the amplified state. To make this clear, we have rearranged the section to make that point first, before discussing additional possibilities. Although the process of centriole amplification and cell division must be of high enough fidelity to produce enough OSNs for the olfactory epithelium, we also agree that mitotic issues may occur with some frequency, and the ability to tolerate this is not well-characterized in differentiated tissues. We leave this possibility open, particularly in our reference to work by Cosenza et al. (lines 283-286). These changes are reflected in the third paragraph of the Discussion section.

*Point 8. Line 166-167: Centriolar rosettes and single centrioles in OSNs have been documented before and citation would be appropriate*.

To our knowledge, single centrioles have not been documented to be present in rosette-bearing cells of the olfactory epithelium, and this is one novel aspect of the work we present here. We have, however, added another reference to work by Cuschieri and Bannister, 1975, to the Discussion section (line 246) as well as in the Introduction and Results.

*Point 9. A scheme, Figure 4. It reads like there is a direct centriole amplification from one mitosis to the next, one interphase should be added in between*.

Thank you for the suggestion. We’ve added “interphase” to the arrow between the two mitoses to convey that cells don’t proceed directly from one mitosis to the next.

Reviewer #3:

*Minor issue: There is only one point I would like to suggest to authors, which is to examine when/how these centrioles that are produced in cycling cells eventually acquire the appendages for ciliogenesis. Appendage formation is one of the final steps in centriole maturation process that in cycling cells normally takes ~1.5 cell cycle to finish (i.e. must go through two runs of mitosis), but in postmitotic cells, it can happen in the same cell cycle phase in which centrioles are born. It will be intriguing to know if centrioles inherited by OSNs also need to go through two or just one run of mitosis to be fully matured. Perhaps the authors can separately check NeuroD+ and NeuroD- cells carrying numerous centrioles for distal appendage markers? It could be interesting but not necessary for the paper as is*.

We agree that this is an interesting consideration and thank the reviewer for the suggestion. Unfortunately, immunofluorescent staining with antibodies against distal appendage proteins hasn’t yielded sufficiently good signal to noise in our hands yet. New techniques (see response to Reviewer 2) and further optimization might provide insight in the future; however, the time required to optimize puts these efforts beyond what we could present here.
